# Acute kidney injury development is associated with mortality in Japanese patients with cirrhosis: impact of amino acid imbalance

**DOI:** 10.1007/s00535-024-02126-7

**Published:** 2024-06-11

**Authors:** Takao Miwa, Yuki Utakata, Tatsunori Hanai, Masashi Aiba, Shinji Unome, Kenji Imai, Koji Takai, Makoto Shiraki, Naoki Katsumura, Masahito Shimizu

**Affiliations:** 1 Department of Gastroenterology/Internal Medicine, Graduate School of Medicine, Gifu University, 1-1 Yanagido, Gifu, 501-1194 Japan; 2 Department of Gastroenterology, Chuno Kosei Hospital, Gifu, Japan

**Keywords:** Acute kidney injury, Amino acid imbalance, Branched-chain amino acids, Hepatorenal syndrome, Survival

## Abstract

**Background:**

Acute kidney injury (AKI) is a serious complication of cirrhosis. This study analyzed the prognostic effect of AKI in patients with cirrhosis and its risk factors, particularly in relation to amino acid imbalance.

**Methods:**

This retrospective study reviewed 808 inpatients with cirrhosis at two institutes in Gifu, Japan. AKI was diagnosed according to the recommendations of the International Club of Ascites. Amino acid imbalance was assessed by measuring serum branched-chain amino acid (BCAA) levels, tyrosine levels, and the BCAA-to-tyrosine ratio (BTR). Factors associated with mortality and AKI development were assessed using the Cox proportional hazards regression model with AKI as a time-dependent covariate and the Fine–Gray competing risk regression model, respectively.

**Results:**

Of the 567 eligible patients without AKI at baseline, 27% developed AKI and 25% died during a median follow-up period of 4.7 years. Using a time-dependent covariate, AKI development (hazard ratio [HR], 6.25; 95% confidence interval [CI], 3.98–9.80; *p* < 0.001) was associated with mortality in patients with cirrhosis independent of potential covariates. In addition, alcohol-associated/-related liver disease, metabolic dysfunction-associated steatohepatitis, Child–Pugh score, and BTR (subdistribution HR 0.78; 95% CI 0.63–0.96; *p* = 0.022) were independently associated with AKI development in patients with cirrhosis. Similar results were obtained in the multivariate model that included BCAA and tyrosine levels instead of BTR.

**Conclusions:**

AKI is common and associated with mortality in Japanese patients with cirrhosis. An amino acid imbalance is strongly associated with the development of AKI in patients with cirrhosis.

**Supplementary Information:**

The online version contains supplementary material available at 10.1007/s00535-024-02126-7.

## Introduction

Acute kidney injury (AKI) is a life-threatening complication of cirrhosis [1, 2]. AKI occurs in approximately 30% of hospitalized patients with cirrhosis, and recent meta-analyses have shown that mortality is six- to sevenfold higher in those with AKI than in those without AKI [3, 4]. However, the association between the development of AKI and the mortality in Japanese patients with cirrhosis and the risk factors for AKI has rarely been studied. While decreased liver functional reserves partly explain the risk of AKI in patients with cirrhosis [3, 5], the evidence is based on cross-sectional or short-term observational studies, and the long-term risk factors for the development of AKI are unclear. Therefore, assessing the prognostic impact of AKI, particularly in Japanese patients with cirrhosis, for whom evidence is scarce, and elucidating the factors associated with the development of AKI are crucial to promote early prevention and treatment in this population.

Patients with cirrhosis have an altered amino acid concentration profile, known as amino acid imbalance, with a decrease in branched-chain amino acids (BCAA), such as leucine, isoleucine, and valine, and an increase in aromatic amino acids, such as phenylalanine, tryptophan, and tyrosine [6]. The BCAA-to-tyrosine ratio (BTR) is an easy-to-use biomarker for assessing amino acid imbalance in patients with cirrhosis [7, 8]. This imbalance is known to be associated with complications of cirrhosis, including hepatic encephalopathy, hepatocellular carcinoma, and sarcopenia [6, 9, 10]. A recent study reported that the assessment of urinary and serum amino acid metabolites, including BCAAs, is useful for predicting the development of AKI in hospitalized patients with cirrhosis [11]. However, the relationship between amino acid imbalance and AKI development in patients with cirrhosis has been little studied, especially in longitudinal studies.

This study aimed to investigate the incidence of AKI and its effects on mortality in Japanese patients with cirrhosis. The secondary aim was to elucidate the factors associated with AKI development in patients with cirrhosis by assessing amino acid imbalances.

## Methods

### Study protocol

This retrospective cohort study included 808 Japanese patients with cirrhosis treated at Gifu University Hospital (*n* = 692) and Chuno Kosei Hospital (*n* = 116). The study protocol was approved by the Institutional Review Board of Gifu University Graduate School of Medicine (approval number: 2023–316). This study was conducted in accordance with the principles of the 2013 Declaration of Helsinki. Owing to the retrospective nature of the study, informed consent was obtained using an opt-out method.

### Participants and follow-up

The study enrolled patients aged ≥ 18 years with any etiology of cirrhosis who were admitted between March 2004 and March 2022. Cirrhosis was diagnosed based on clinical characteristics including complications, liver histology, laboratory data, and medical imaging. The exclusion criteria were organ transplant including the liver, active malignancies including hepatocellular carcinoma (HCC), a history of AKI within 1 month, estimated glomerular filtration rate (eGFR) < 15 mL/min/1.73 m^2^, dialysis, overt hepatic encephalopathy (OHE) or BCAA infusion within 1 month, life-threatening comorbidities, and opt-out refusal. After discharge, patients were monitored at least every three months at the outpatient clinic based on the Japanese guidelines for cirrhosis [12, 13].

### Diagnostic criteria and staging of AKI

Diagnosis and staging of AKI were based on the consensus recommendations of the International Club of Ascites [1, 2]. The serum creatinine (sCr) value obtained in the previous three months or the value closest to admission was used as the baseline sCr. AKI was diagnosed if sCr increased ≥ 0.3 mg/dl within 48 h or ≥ 50% from baseline, and the increase was known or suspected to have occurred within 7 days. In addition, AKI stages were categorized as follows: stage 1 as an increase in sCr ≥ 0.3 mg/dl or ≥ 1.5- to twofold from baseline, stage 2 as an increase in sCr > 2- to threefold from baseline, and stage 3 as an increase in sCr ≥ threefold from baseline or sCr ≥ 4.0 mg/dl with an acute increase of ≥ 0.3 mg/dl or initiation of renal replacement therapy [1, 2]. The outcomes were followed-up until the last visit, death, or December 25, 2023, whichever occurred first.

### Data collection

The following baseline data were collected from the medical records: age; sex; weight; height; etiology of cirrhosis; comorbidities including diabetes mellitus, hypertension, heart failure, ascites, and hepatic encephalopathy; laboratory data; and medications including use of BCAA infusion. Serum concentrations of BCAA, tyrosine, and BTR were measured under overnight fasting conditions by SRL, Inc., Tokyo, Japan. BTR ≤ 4.4, BCAA < 344 μmol/L, and tyrosine > 99 μmol/L were defined as low BTR, low BCAA, and high tyrosine, respectively, based on the reference data [7, 8]. Body mass index (BMI), Child–Pugh score, model for end-stage liver disease (MELD) score, and eGFR were automatically calculated from the obtained data. Demographic variables were assessed at the time of admission and biochemical parameters were evaluated on the day of admission or the following day under fasting condition. Regarding outcomes, the dates of AKI, OHE, HCC, and death were recorded, and the time to each event was calculated using the date of enrollment. HCC was assessed using medical images and OHE was assessed based on the West Haven criteria [14].

### Statistical analysis

Data are expressed as medians with interquartile ranges for continuous variables and as numbers with percentages for categorical variables. The baseline characteristics of the groups were compared using the Mann–Whitney *U* test or chi-square test. Survival curves were estimated using the Kaplan–Meier method, and groups were compared using the log-rank test. Factors associated with survival were assessed using the Cox proportional hazards regression model, and the results were expressed as hazard ratios (HRs) with 95% confidence intervals (CIs). Multiple comparisons were performed using the Bonferroni correlation test. Multivariate analysis was performed using AKI, OHE, and HCC development as time-dependent covariates to examine the association between AKI development and mortality [15]. Considering mortality as a competing risk, the cumulative incidence curves of AKI were estimated using the cumulative incidence function, and the groups were compared using the Gray’s test. Factors associated with AKI development were investigated using the Fine–Gray competing risk regression model, and the results were presented as subdistribution hazard ratios (SHRs) with 95% CIs. As the main exposure of interest was amino acid imbalance, multivariate analyses were performed by including BTR in Model 1 and serum BCAA and tyrosine levels in Model 2. Furthermore, factors associated with amino acid imbalance was assessed using the logistic regression model, and the results were demonstrated as odds ratios with 95% CIs. Covariate selection was prespecified to avoid overfitting.

A two-sided *p* < 0.05 was set as the threshold for statistical significance. All statistical analyses were performed using the R software, version 4.3.2 (The R Foundation for Statistical Computing, Vienna, Austria).

## Results

### Clinical characteristics of enrolled patients with cirrhosis

Of 808 screened patients, 567 met the eligibility criteria and were included in the analysis (Supplementary Fig. 1). The baseline characteristics of the included patients are shown in Table 1. The median age of the 567 patients was 67 years, 50% were male, and the median BMI was 23.1 kg/m^2^. Ascites was present in 34% of the patients, with a median serum creatinine of 0.71 mg/dL. The predominant etiology of cirrhosis was viral hepatitis (40%), followed by alcohol-associated/related liver disease (ALD) (22%), metabolic dysfunction-associated steatohepatitis (MASH) (10%), and other causes (27%). The median Child–Pugh and MELD scores were 6 and 8, respectively. Assessment of amino acid concentrations showed a median BCAA level of 379 μmol/L, tyrosine level of 90 μmol/L, and BTR of 4.31. Regarding medications which can cause AKI, none of them took adefovir, 1% took tenofovir, and 3% received cisplatin due to HCC development.

**Table 1:** Baseline characteristics of patients with cirrhosis according to AKI

Characteristic	All patients	No AKI	AKI	*p-*value*
	(*n* = 567)	(*n* = 415)	(*n* = 152)	
Age (years)	67 (57–75)	68 (57–75)	66 (59–73)	0.313
Male, *n* (%)	283 (50)	192 (46)	91 (60)	0.006
Body mass index (kg/m^2^)	23.1 (21.1–25.3)	23.4 (21.5–25.6)	22.1 (20.6–24.5)	0.003
Etiology, *n* (%)				< 0.001
Viral	229 (40)	182 (44)	47 (31)	
ALD	126 (22)	69 (17)	57 (38)	
MASH	58 (10)	47 (11)	11 (7)	
Others	154 (27)	117 (28)	37 (23)	
Diabetes mellitus, *n* (%)	150 (26)	116 (28)	34 (22)	0.264
Hypertension, *n* (%)	172 (30)	127 (22)	45 (30)	0.900
Heart failure, *n* (%)	28 (5)	12 (3)	16 (11)	< 0.001
Ascites, *n* (%)	191 (34)	107 (26)	84 (55)	< 0.001
Child–Pugh score	6 (5–8)	5 (5–7)	8 (6–9)	< 0.001
Child–Pugh class (A/B/C)	334/154/79	282/92/41	52/62/38	< 0.001
MELD score	8 (7–11)	8 (7–10)	10 (8–13)	< 0.001
International normalized ratio	1.08 (1.00–1.22)	1.05 (0.98–1.17)	1.16 (1.06–1.31)	< 0.001
Platelet (10^9^/L)	101 (59–156)	111 (67–162)	75 (41–121)	< 0.001
Blood urea nitrogen (mg/dL)	15 (12–19)	15 (12–18)	15 (12–22)	0.398
Creatinine (mg/dL)	0.71 (0.58–0.87)	0.69 (0.56–0.86)	0.73 (0.63–0.97)	0.005
eGFR (mL/min/1.73 m^2^)	75 (59–91)	77 (60–91)	70 (58–92)	0.109
Albumin (g/dL)	3.5 (2.8–3.9)	3.6 (3.1–4.0)	2.9 (2.4–3.4)	< 0.001
Bilirubin (mg/dL)	1.0 (0.8–1.5)	1.0 (0.7–1.4)	1.2 (0.9–2.2)	< 0.001
Sodium (meq/L)	139 (137–140)	139 (137–140)	138 (136–140)	0.035
Ammonia (μg/dL)	57 (40–79)	53 (38–73)	60 (49–82)	< 0.001
Zinc (μg/dL)	73 (58–85)	75 (62–85)	60 (49–82)	0.001
BTR	4.31 (3.04–5.50)	4.70 (3.53–5.80)	3.14 (2.57–4.38)	< 0.001
BCAA (μmol/L)	379 (311–453)	393 (336–467)	322 (264–390)	< 0.001
Tyrosine (μmol/L)	90 (71–113)	88 (69–109)	100 (83–125)	< 0.001
Medications				
BCAA, *n* (%)	223 (39)	134 (32)	89 (59)	< 0.001
Rifaximin, *n* (%)	37 (7)	20 (5)	17 (11)	0.012
Nonabsorbable disaccharides, *n* (%)	65 (11)	40 (10)	25 (16)	0.035
Zinc, *n* (%)	45 (8)	30 (7)	15 (10)	0.303
Levocarnitine, *n* (%)	36 (6)	21 (5)	15 (10)	0.038
Diuretics, *n* (%)	202 (36)	121 (29)	81 (53)	< 0.001
Cisplatin, *n* (%)	17 (3)	11 (3)	6 (4)	0.600
Tenofovir, *n* (%)	7 (1)	7 (2)	0 (0)	0.237

Values are presented as numbers (percentages) or medians (interquartile range)

*Statistical differences between two groups were analyzed using the chi-square test or Mann–Whitney *U* test

AKI, acute kidney injury; ALD, alcohol-associated/related liver disease; BCAA, branched-chain amino acid; BTR, branched-chain amino acid-to-tyrosine ratio; eGFR, estimated glomerular filtration rate; MASH, metabolic dysfunction-associated steatohepatitis; MELD, model for end-stage liver disease

### Comparison of patients with and without AKI development

As shown in Table 1, patients who developed AKI were more likely to be male, and the etiology of cirrhosis was significantly different from that of patients without AKI. In addition, patients who developed AKI had worse liver functional reserves in terms of ascites, Child–Pugh score, MELD score, international normalized ratio, platelet count, and albumin, bilirubin, sodium, ammonia, and zinc levels than those without AKI. Furthermore, patients who developed AKI had lower BMI and higher serum creatinine levels than those without AKI. Patients who developed AKI had a significantly lower BTR (3.14 vs. 4.70; *p* < 0.001), detailed by lower BCAA levels (322 vs. 393 μmol/L; *p* < 0.001) and higher tyrosine levels (100 vs. 88 μmol/L; *p* < 0.001) than those without AKI.

### Incidence of AKI and other events in patients with cirrhosis

During a median follow-up period of 4.7 years (interquartile range, 1.1–5.5), 27% (*n* = 152) of patients with cirrhosis developed AKI. Of these, 66% (*n* = 101) were in stage 1, 19% (*n* = 29) were in stage 2, and 14% (*n* = 22) were in stage 3. The overall incidence rates of AKI at 1, 3, and 5 years were 14%, 23%, and 29%, respectively.

Regarding other events, OHE was developed in 16% (*n* = 91) and 73% (*n* = 66) were in grade 2, 20% (*n* = 18) were in stage 3, 4% (*n* = 4) were in grade 4, and 3% (*n* = 3) were in stage 5. The overall incidence rates of OHE at 1, 3, and 5 years were 7%, 12%, and 16%, respectively. HCC was detected in 13% (*n* = 73) and the overall incidence rates of at 1, 3, and 5 years were 3%, 10%, and 12%, respectively. Among the development of AKI, OHE, and HCC, 2% (*n* = 13) experienced all three events, 11% (*n* = 64) experienced two events, and 26% (*n* = 149) experienced one event.

### Association between AKI development and mortality

During the follow-up period, 25% (*n* = 139) of the patients died of liver failure (*n* = 96; 69%), hepatocellular carcinoma (*n* = 17; 12%), and other causes (*n* = 26; 19%). The 1-, 3-, and 5-year overall survival rates were 90%, 80%, and 74%, respectively. The adjusted HRs for the factors associated with mortality, considering AKI and OHE as time-dependent covariates, are presented in Table 2. When analyzed using time-dependent covariates, AKI development (HR 6.25; 95% CI 3.98–9.80; *p* < 0.001) was significantly associated with mortality independent of the etiology of cirrhosis, Child–Pugh score, OHE development, and HCC development. Details of the multivariate analyses are shown in Supplementary Table 1.

**Table 2:** Adjusted HRs for factors related to mortality, including time-dependent covariates, in patients with cirrhosis

Characteristic	HR (95% CI)	*p*-value^*^
Baseline covariates		
Etiology of cirrhosis		
Viral^a^	1.00	
ALD	1.63 (1.01–2.63)	0.047
MASH	0.59 (0.15–1.81)	0.311
Others	1.25 (0.73–2.14)	0.409
Child–Pugh score	1.17 (1.05–1.29)	0.003
Time dependent covariates		
AKI development	6.25 (3.98–9.80)	< 0.001
OHE development	8.81 (2.68–13.68)	< 0.001
HCC development	3.10 (2.01–4.81)	< 0.001

^*^Multivariate analysis using the Cox proportional hazards model, including age, sex, body mass index, etiology, diabetes mellitus, Child–Pugh score, creatinine, and sodium as baseline covariates, and AKI, OHE, and HCC as time-dependent covariates

^a^Reference group

Abbreviations: AKI, acute kidney injury; ALD, alcohol-associated/related liver disease; CI, confidence interval; HCC, hepatocellular carcinoma; HR, subdistribution hazard ratio; MASH, metabolic dysfunction-associated steatohepatitis; OHE, overt hepatic encephalopathy

**Fig. 1:**
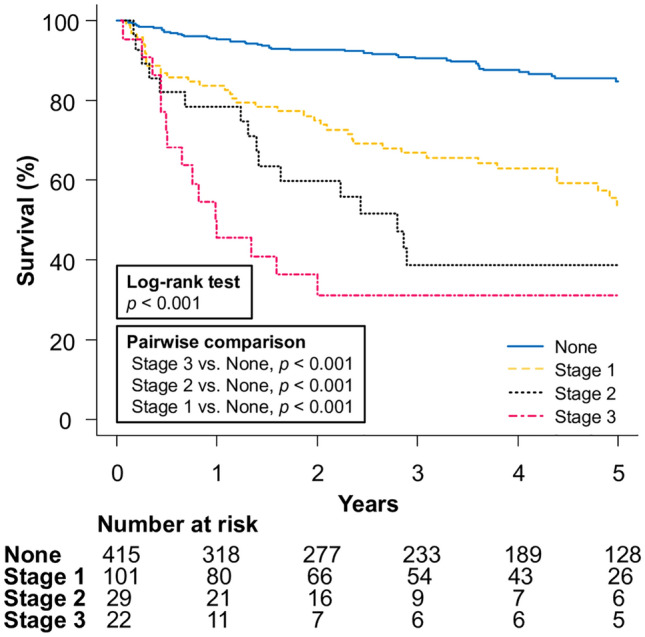
Overall survival of patients with cirrhosis by stage of acute kidney injury

Cox proportional hazards regression also showed that patients who developed AKI stage 1 (HR 3.94; 95% CI 2.67–5.18; *p* < 0.001), stage 2 (HR 5.83; 95% CI 3.41–9.96; *p* < 0.001), and stage 3 (HR 9.39; 95% CI 5.55–15.90; *p* < 0.001) had significantly higher mortality than those without AKI development. The survival curve showed that patients who developed advanced AKI had significantly worse survival rates than those in earlier stages (Fig. 1, *p* < 0.001).

### Factors associated with AKI development

Table 3 shows the adjusted SHRs for factors associated with AKI development. Multivariate Model 1 showed that ALD (SHR 2.12; 95% CI, 1.16–3.87; *p* = 0.014), MASH (SHR 2.72; 95% CI 1.22–6.06; *p* = 0.014), Child–Pugh score (SHR 1.24; 95% CI 1.03–1.49; *p* = 0.020), and BTR (SHR 0.78; 95% CI 0.63–0.96; *p* = 0.022) were independently associated with AKI development in patients with cirrhosis. In Model 2, BCAA (SHR 1.00; 95% CI 0.99–1.00; *p* = 0.031) and tyrosine (SHR 1.01; 95% CI 1.00–1.01; *p* = 0.045) were similarly associated with AKI development. Details of the multivariate analyses are shown in Supplementary Table 2. In addition, factors associated with amino acid imbalance (BTR ≤ 4.4) are shown in Supplementary Table 4.

**Table 3:** Adjusted SHRs for factors associated with AKI development in patients with cirrhosis

	Model 1^a^			Model 2^b^	
Characteristic	SHR (95% CI)	*p*-value^*^		SHR (95% CI)	*p*-value^*^
Etiology of cirrhosis					
Viral^c^	1.00			1.00	
ALD	2.12 (1.16–3.87)	0.014		2.09 (1.15–3.82)	0.016
MASH	2.72 (1.22–6.06)	0.014		2.54 (1.19–5.40)	0.016
Others	1.17 (0.62–2.21)	0.630		1.15 (0.61–2.18)	0.670
Child–Pugh score	1.24 (1.03–1.49)	0.020		1.24 (1.03–1.49)	0.026
BTR	0.78 (0.63–0.96)	0.022			
BCAA (μmol/L)				1.00 (0.99–1.00)	0.031
Tyrosine (μmol/L)				1.01 (1.00–1.01)	0.045

^*^Multivariate analyses were performed using the Fine–Gray competing risk regression model

^a^Model 1 included age, sex, body mass index, etiology, diabetes mellitus, hypertension, heart failure, Child–Pugh score, and blood urea nitrogen, creatinine, ammonia, zinc, BTR levels, tenofovir use, and cisplatin use

^b^Model 2 included age, sex, body mass index, etiology, diabetes mellitus, Child–Pugh score, and blood urea nitrogen, creatinine, ammonia, zinc, BCAA, tyrosine levels, tenofovir use, and cisplatin use

^c^Reference group

Abbreviations: AKI, acute kidney injury; ALD, alcohol-associated/related liver disease; BCAA, branched-chain amino acid; BTR, branched-chain amino acid-to-tyrosine ratio; CI, confidence interval; MASH, metabolic dysfunction-associated steatohepatitis; SHR, subdistribution hazard ratio

**Fig. 2:**
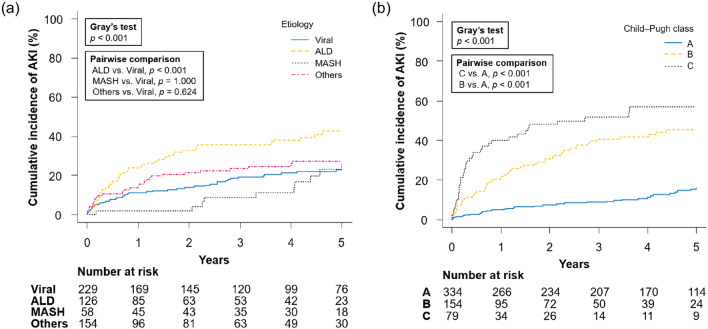
Cumulative incidence of AKI in patients with cirrhosis according to **a** etiology of cirrhosis and **b** Child–Pugh class. *AKI* acute kidney injury, *ALD* alcohol-associated/related liver disease, *MASH* metabolic dysfunction-associated steatohepatitis

**Fig. 3:**
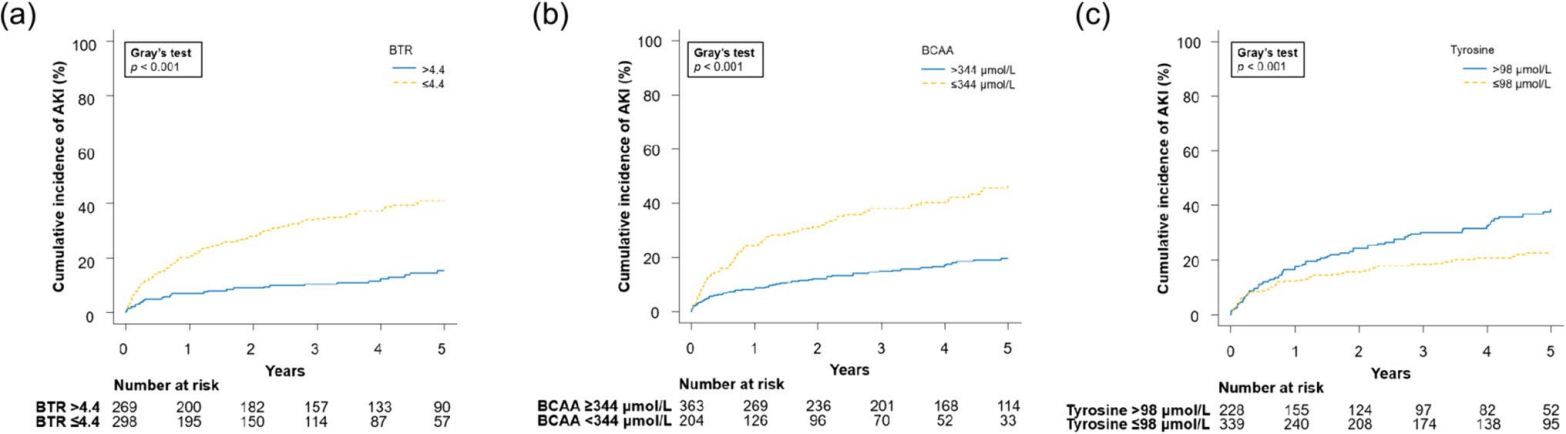
Cumulative incidence of AKI in patients with cirrhosis according to **a** BTR ≤ 4.4 and > 4.4, **b** BCAA < 344 μmol/L and ≥ 344 μmol/L, and **c** tyrosine > 98 μmol/L and ≤ 98 μmol/L. *AKI* acute kidney injury, *BCAA* branched-chain amino acid, *BTR* branched-chain amino acid-to-tyrosine ratio

The cumulative incidences of AKI at 1, 3, and 5 years were 11%, 19%, and 23%, respectively, for viral hepatitis; 24%, 36%, and 43%, respectively, for ALD; 2%, 9%, and 27%, respectively, for MASH; and 15%, 23%, and 27%, respectively, for other causes (*p* < 0.001; Fig. 2a). Furthermore, the cumulative incidence of AKI at 1, 3, and 5 years was 5%, 9%, and 16% for Child–Pugh class A; 22%, 41%, and 45% for class B; and 40%, 52%, and 57% for class C, respectively (*p* < 0.001; Fig. 2b). Regarding amino acid imbalance, the cumulative incidence of AKI was significantly higher in patients with BTR ≤ 4.4 than in those with BTR > 4.4 (21%, 34%, and 42% vs. 7%, 10%, and 15% at 1, 3, and 5 years, respectively; *p* < 0.001; Fig. 3a), in patients with BCAA < 344 μmol/L than in those with BCAA ≥ 344 μmol/L (24%, 38%, and 47% vs. 9%, 15%, and 20% at 1, 3, and 5 years, respectively; *p* < 0.001; Fig. 3b), and in patients with tyrosine ≥ 98 μmol/L than in those with tyrosine < 98 μmol/L (18%, 30%, and 39% vs. 12%, 18%, and 23% at 1, 3, and 5 years, respectively; *p* < 0.001; Fig. 3c).

### Association between of medications for cirrhosis and AKI development

Multivariate model to assess the relationship between medications for cirrhosis and AKI development is shown in Supplementary Table 3. The results revealed that diuretics (SHR 2.35; 95% CI 1.25–4.40; *p* = 0.008) and nonabsorbable disaccharides (SHR 0.41; 95% CI 0.17–0.97; *p* = 0.042) were associated with AKI development, while BCAA supplementation and rifaximin were not statistically significant.

## Discussion

AKI is a devastating complication of cirrhosis and biomarkers that can predict its development are urgently needed. However, few longitudinal studies have been conducted in patients without AKI at baseline. Our study revealed that AKI development was associated with mortality in Japanese patients with cirrhosis. Furthermore, we elucidated the factors associated with AKI development in patients with cirrhosis and demonstrated that amino acid imbalance was robustly associated with AKI development in these patients.

The first important finding of our study was the prognostic significance of AKI in Japanese patients with cirrhosis. AKI development is an established risk factor for mortality in patients with cirrhosis [3, 4]. However, the effect of AKI on mortality in Japanese patients with cirrhosis has yet to be explored. Therefore, the results of this study showing the prognostic effect of AKI in patients with cirrhosis are of great clinical importance. The use of time-dependent covariates is an established method for investigating the association between event occurrence and outcomes [15]. Using this technique, we found that AKI development was associated with mortality in patients with cirrhosis who had no AKI at baseline. Furthermore, the HRs for mortality in patients with cirrhosis increased with increasing AKI stage, similar to the results of a multicenter study in the US [16]. As reversing AKI improves outcomes in patients with cirrhosis, early detection and intervention is important to reduce mortality [16]. Given the prognostic value of AKI, strategies to identify high-risk groups for AKI development are required to provide early intervention for these patients.

The second integrative finding was the identification of the incidence of and risk factors for AKI development in patients with cirrhosis. The one-year incidence of AKI in Japanese patients with cirrhosis was approximately 14%, which was lower than that reported in the US (21–38%), possibly because of the relatively preserved liver functional reserves in our cohort [11, 17]. A previous meta-analysis showed that worse Child–Pugh class and MELD score are established risk factors for AKI in patients with cirrhosis [3]. Similarly, our study also found a robust association between liver functional reserves and AKI development, with a worse Child–Pugh class being associated with a higher incidence of AKI. In addition, patients with ALD or MASH are at higher risk of developing AKI than those with viral hepatitis. Our findings are supported by previous research suggesting a robust association between alcohol consumption and AKI [18, 19]. Furthermore, patients with ALD are known to have worse mortality than other etiologies [20], which was also confirmed in our study. Renal vascular impairment due to metabolic abnormalities complicated by MASH may also increase the risk of AKI, and nonalcoholic fatty liver disease has been reported as a risk factor for AKI readmission in patients with heart failure [21]. Given the increasing prevalence of ALD and MASH according to recent epidemiological data [22], our results suggest that AKI is a more frequent burden in patients with cirrhosis.

The present study also found a strong association between amino acid imbalance, as assessed by serum BCAA and tyrosine levels, BTR, and AKI development in patients with cirrhosis. While previous studies have documented the association between amino acid imbalance and hepatic encephalopathy [6], limited evidence is available regarding its association with AKI. A recent multicenter study assessing serum and urine metabolites reported that aromatic and BCAA metabolisms are major contributors to AKI development in patients with cirrhosis [11]. The present study also highlighted the negative influence of amino acid imbalance on AKI development, especially the independent impact of increased tyrosine and decreased BCAA levels, which are protective of the liver and against complications of cirrhosis [23, 24]. Therefore, this longitudinal study expands the existing knowledge and strengthens the evidence of the association between amino acid imbalance and AKI development in patients with cirrhosis.

As for medications, use of diuretics and nonabsorbable disaccharides were associated with AKI, while other protective medications were not in our study. BCAA supplementation improves serum albumin levels, which are important for maintaining circulating plasma volume. Furthermore, rifaximin improves gut barrier function and a retrospective study has reported a protective effect on AKI in patients with cirrhosis [25, 26]. However, patients receiving liver-protective medications, such as BCAA or rifaximin, exhibited a significantly higher incidence of AKI compared to those not receiving these treatments in our study. This discrepancy may be due to the reduced liver functional reserves in patients with these medications than those without. In addition, diuretics for ascites, medications for HCC, and oral nucleoside/nucleotide analogs for hepatitis B can trigger AKI in patients with cirrhosis [27, 28]. Since strategies to prevent AKI development are not well-established, future research should evaluate the efficacy of medications for cirrhosis, including BCAA supplementation and rifaximin treatment, in preventing AKI in patients with cirrhosis.

This study has several limitations. First, it was a study of patients with cirrhosis in Gifu, Japan, which may limit the generalizability of the results to other regions. Second, owing to the retrospective nature of the study, we did not evaluate the specific causes of AKI because it was difficult to identify the cause of AKI with the available data. Third, the incidence of AKI development may have varied according to the timing of the biochemical assessment. Fourth, the limited number of patients with MASH may have affected the results of our study. Despite these limitations, the study has notable strengths, including an adequate sample size, a substantial number of events for robust analysis, and the application of a gold-standard definition for the assessment of AKI in patients with liver cirrhosis [1, 2].

In conclusion, our study demonstrated the incidence and prognostic value of AKI in Japanese patients with cirrhosis. Furthermore, our study highlights the strong association between amino acid imbalances and AKI development in these patients. Future research is warranted to validate our findings and establish effective strategies for the prevention and treatment of AKI from the perspective of amino acid imbalances.

### Supplementary Information

Below is the link to the electronic supplementary material.

Supplementary file1 (DOCX 162 KB)
