# Supplementary material for: Acute kidney injury development is associated with mortality in Japanese patients with cirrhosis: impact of amino acid imbalance
Source: J Gastroenterol. 2024 Jun 11;59(9):849–57. doi: 10.1007/s00535-024-02126-7 (PMC11338968; doi:10.1007/s00535-024-02126-7)
Supplement: Supplementary file 1 — Supplementary file1 (DOCX 162 KB) [file 535_2024_2126_MOESM1_ESM.docx]

**Acute kidney injury development is associated with mortality in Japanese patients with cirrhosis: Impact of amino acid imbalance**

Takao Miwa^1*^, Yuki Utakata^2^, Tatsunori Hanai^1^, Masashi Aiba^2^, Shinji Unome^1^, Kenji Imai^1^, Koji Takai^1^, Makoto Shiraki^2^, Naoki Katsumura^2^, and Masahito Shimizu^1^

1) Department of Gastroenterology/Internal Medicine, Graduate School of Medicine, Gifu University, Gifu, Japan

2) Department of Gastroenterology, Chuno Kosei Hospital, Gifu, Japan

***Corresponding author:** Takao Miwa M.D., Ph.D.

Department of Gastroenterology/Internal Medicine, Graduate School of Medicine, Gifu University, 1-1 Yanagido, Gifu 501-1194, Japan

Tel: +81-58-230-6308

Fax: +81-58-230-6310

E-mail: [miwa.takao.a6@f.gifu-u.ac.jp](mailto:miwa.takao.a6@f.gifu-u.ac.jp)

**Supplementary Table 1.** Details of the multivariate analysis of factors related to mortality, including time-dependent covariates, in patients with cirrhosis

| Characteristic | HR (95% CI) | *p*-value^*^ |
| --- | --- | --- |
| Baseline covariates |  |  |
| Age (years) | 1.02 (1.00–1.04) | 0.080 |
| Male | 1.18 (0.71–1.97) | 0.519 |
| Body mass index (kg/m^2^) | 1.00 (0.95–1.06) | 0.902 |
| Etiology of cirrhosis |  |  |
| Viral^a^ | 1.00 |  |
| ALD | 1.63 (1.01–2.63) | 0.047 |
| MASH | 0.59 (0.15–1.81) | 0.311 |
| Others | 1.25 (0.73–2.14) | 0.409 |
| Diabetes mellitus | 0.76 (0.49–1.17) | 0.212 |
| Child-Pugh score | 1.17 (1.05–1.29) | 0.003 |
| Creatinine (mg/dL) | 0.57 (0.29–1.10) | 0.092 |
| Sodium (meq/L) | 0.97 (0.91–1.04) | 0.389 |
| Time-dependent covariates |  |  |
| AKI development | 6.25 (3.98–9.80) | <0.001 |
| OHE development | 8.81 (2.68–13.68) | <0.001 |
| HCC development | 3.10 (2.01–4.81) | <0.001 |

*Multivariate analyses were performed using the Cox proportional hazard model.

^a^Reference group

Abbreviations: AKI, acute kidney injury; ALD, alcohol-associated/related liver disease; CI, confidence interval; HCC, hepatocellular carcinoma; HR, subdistribution hazard ratio; MASH, metabolic dysfunction-associated steatohepatitis; OHE, overt hepatic encephalopathy

**Supplementary Table 2.** Details of the multivariate analyses of factors related to AKI development in patients with cirrhosis

|  | Model 1 | |  | Model 2 | |
| --- | --- | --- | --- | --- | --- |
| Characteristic | SHR (95% CI) | *p*-value^*^ |  | SHR (95% CI) | *p*-value^*^ |
| Age (years) | 1.01 (0.99–1.03) | 0.410 |  | 1.01 (0.99–1.03) | 0.260 |
| Male | 1.48 (0.88–2.48) | 0.140 |  | 1.59 (0.94–2.68) | 0.084 |
| Body mass index (kg/m^2^) | 0.94 (0.89–1.00) | 0.065 |  | 0.95 (0.90–1.01) | 0.130 |
| Etiology of cirrhosis |  |  |  |  |  |
| Viral^a^ | 1.00 |  |  | 1.00 |  |
| ALD | 2.12 (1.16–3.87) | 0.014 |  | 2.09 (1.15–3.82) | 0.016 |
| MASH | 2.72 (1.22–6.06) | 0.014 |  | 2.54 (1.19–5.40) | 0.016 |
| Others | 1.17 (0.62–2.21) | 0.630 |  | 1.15 (0.61–2.18) | 0.670 |
| Diabetes mellitus | 0.57 (0.31–1.05) | 0.073 |  | 0.59 (0.32–1.10) | 0.095 |
| Hypertension | 1.00 (0.58–1.75) | 0.990 |  | 0.99 (0.57–1.75) | 0.980 |
| Heart failure | 4.42 (2.01–9.71) | <0.001 |  | 4.33 (1.96–9.59) | <0.001 |
| Child–Pugh score | 1.24 (1.03–1.49) | 0.020 |  | 1.24 (1.03–1.49) | 0.026 |
| Blood urea nitrogen (mg/dL) | 1.02 (1.01–1.03) | 0.003 |  | 1.02 (1.01–1.03) | 0.001 |
| Creatinine (mg/dL) | 1.24 (0.75–2.04) | 0.410 |  | 1.07 (0.64–1.81) | 0.790 |
| Ammonia (μg/dL) | 1.00 (0.99–1.01) | 0.870 |  | 1.00 (0.99–1.01) | 0.870 |
| Zinc (μg/dL) | 1.01 (1.00–1.02) | 0.220 |  | 1.01 (1.00–1.02) | 0.100 |
| BTR | 0.78 (0.63–0.96) | 0.022 |  |  |  |
| BCAA (μmol/L) |  |  |  | 1.00 (0.99–1.00) | 0.031 |
| Tyrosine (μmol/L) |  |  |  | 1.01 (1.00–1.01) | 0.045 |
| Medications |  |  |  |  |  |
| Tenofovir | NA | NA |  | NA | NA |
| Cisplatin | 0.37 (0.08–1.68) | 0.200 |  | 0.35 (0.07–1.68) | 0.190 |

*Multivariate analyses were performed using the Fine–Gray competing risk regression model.

^a^Reference group

Abbreviations: AKI, acute kidney injury; ALD, alcohol-associated/related liver disease; BCAA, branched-chain amino acid; BTR, branched-chain amino acid to tyrosine ratio; CI, confidence interval; MASH, metabolic dysfunction-associated steatohepatitis; NA, not available; SHR, sub-distribution hazard ratio

**Supplementary Table 3.** Multivariate analysis to assess the relationship between medications for cirrhosis and AKI development

| Characteristic | SHR (95% CI) | *p*-value^*^ |
| --- | --- | --- |
| Age (years) | 1.00 (0.98–1.02) | 0.830 |
| Male | 1.54 (0.87–2.74) | 0.140 |
| Body mass index (kg/m^2^) | 0.94 (0.88–1.00) | 0.044 |
| Etiology of cirrhosis |  |  |
| Viral^a^ | 1.00 |  |
| ALD | 2.15 (1.12–4.15) | 0.022 |
| MASH | 2.03 (0.79–5.26) | 0.140 |
| Others | 1.26 (0.67–2.39) | 0.470 |
| Diabetes mellitus | 0.48 (0.25–0.91) | 0.026 |
| Hypertension | 1.21 (0.70–2.10) | 0.500 |
| Heart failure | 4.89 (2.19–10.94) | <0.001 |
| Child–Pugh score | 1.12 (0.93–1.35) | 0.250 |
| Blood urea nitrogen (mg/dL) | 1.02 (1.01–1.03) | 0.002 |
| Creatinine (mg/dL) | 1.15 (0.65–2.02) | 0.630 |
| Ammonia (μg/dL) | 1.00 (0.99–1.01) | 0.660 |
| Zinc (μg/dL) | 1.01 (0.99–1.01) | 0.310 |
| BTR | 0.78 (0.65–0.96) | 0.019 |
| Medications |  |  |
| BCAA | 0.86 (0.45–1.65) | 0.650 |
| Rifaximin | 1.54 (0.56–4.22) | 0.400 |
| Nonabsorbable disaccharides | 0.41 (0.17–0.97) | 0.042 |
| Zinc | 1.68 (0.72–3.94) | 0.230 |
| Levocarnitine | 1.42 (0.48–4.22) | 0.530 |
| Diuretics | 2.35 (1.25–4.40) | 0.008 |

*Multivariate analysis was performed using the Fine–Gray competing risk regression model.

^a^Reference group

Abbreviations: AKI, acute kidney injury; ALD, alcohol-associated/related liver disease; BCAA, branched-chain amino acid; BTR, branched-chain amino acid to tyrosine ratio; CI, confidence interval; MASH, metabolic dysfunction-associated steatohepatitis; SHR, subdistribution hazard ratio

**Supplementary Table 4.** Multivariate analysis to assess factors associated with amino acid imbalance (BTR ≤4.4) in patients with cirrhosis

| Characteristic | OR (95% CI) | *p*-value^*^ |
| --- | --- | --- |
| Age (years) | 1.01 (0.99–1.04) | 0.165 |
| Male | 0.37 (0.20–0.68) | 0.001 |
| Body mass index (kg/m^2^) | 1.10 (1.02–1.17) | 0.009 |
| Etiology of cirrhosis |  |  |
| Viral^a^ | 1.00 |  |
| ALD | 2.59 (1.26–5.34) | 0.010 |
| MASH | 0.34 (0.14–0.81) | 0.016 |
| Others | 0.65 (0.35–1.19) | 0.163 |
| Diabetes mellitus | 0.66 (0.37–1.19) | 0.164 |
| Child–Pugh score | 1.12 (0.90–1.40) | 0.309 |
| Creatinine (mg/dL) | 0.45 (0.19–1.09) | 0.078 |
| Ammonia (μg/dL) | 1.03 (1.02–1.04) | <0.001 |
| Zinc (μg/dL) | 0.99 (0.97–1.00) | 0.044 |
| Medications |  |  |
| BCAA | 1.99 (0.95–4.17) | 0.069 |
| Rifaximin | 0.45 (0.10–1.92) | 0.280 |
| Nonabsorbable disaccharides | 3.46 (1.08–11.00) | 0.036 |
| Zinc | 1.06 (0.40–2.81) | 0.899 |
| Levocarnitine | 0.40 (0.08–1.93) | 0.255 |
| Diuretics | 1.00 (0.46–2.16) | 0.999 |

*Multivariate analysis was performed using the logistic regression model.

^a^Reference group

Abbreviations: AKI, acute kidney injury; ALD, alcohol-associated/related liver disease; BCAA, branched-chain amino acid; BTR, branched-chain amino acid to tyrosine ratio; CI, confidence interval; MASH, metabolic dysfunction-associated steatohepatitis; OR, odds ratio


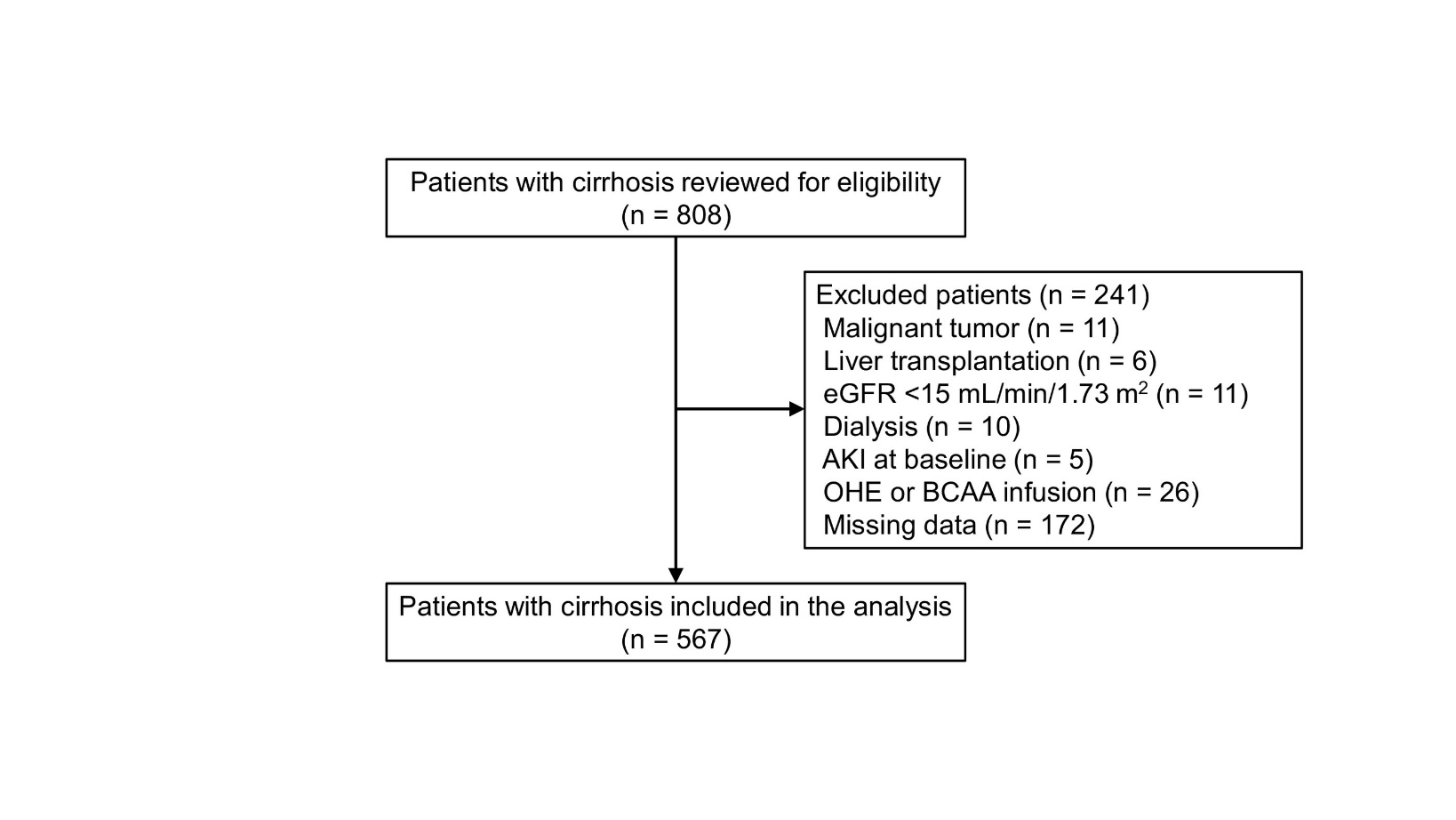
**Supplementary Fig. 1** Flow diagram of the study.

Abbreviations: AKI, acute kidney injury; BCAA, branched-chain amino acid; eGFR, estimated glomerular filtration rate; OHE, overt hepatic encephalopathy
